# Left ventricular longitudinal function assessment in rabbits after acute occlusion of left anterior descending coronary artery by two-dimensional speckle tracking imaging

**DOI:** 10.1186/s12872-017-0655-6

**Published:** 2017-08-08

**Authors:** Jun Huang, Zi-Ning Yan, Li Fan, Yi-Fei Rui, Xiang-Ting Song

**Affiliations:** Department of Echocardiography, ChangZhou No.2 People’s Hospital Affiliated to NanJing Medical University, ChangZhou, 213003 China

**Keywords:** Acute myocardial ischemia, Longitudinal, Rotation, Strain, Strain rate

## Abstract

**Background:**

To evaluate the left ventricular (LV) longitudinal function changes in rabbits after acute occlusion of the left anterior descending artery (LAD) by two-dimensional speckle tracking imaging (2D–STI).

**Methods:**

Forty-eight New Zealand white rabbits underwent echocardiography examination. EchoPAC was used to measure LV peak systolic longitudinal strain (LS) of the endocardium, middle myocardium, and epicardium, peak longitudinal strain rate (LSr), segmental and global longitudinal rotation (LR) degrees. Ligated the LAD and repeated all measurements after 10 min.

**Results:**

Peak LS and LSr were significantly different between the preoperative and postoperative rabbits among most LV walls (*P* < 0.05). In apical four-chamber view, there was significant difference in the degrees of rotation of the LV lateral wall in preoperative and postoperative rabbits (*P* < 0.05). In apical three-chamber view, the rotation degrees of the posterior wall and the LR were significantly lower in the postoperative than in the preoperative (*P* < 0.001). In apical two-chamber view, the rotation degrees of the inferior wall and the LR were significantly lower in the postoperative (*P* < 0.05).

**Conclusions:**

Left ventricular function was impaired after acute occlusion of LAD. Segmental rotational degrees and changes in LR could be useful indicators of cardiac function during the early phases of acute myocardial ischemia.

**Electronic supplementary material:**

The online version of this article (doi:10.1186/s12872-017-0655-6) contains supplementary material, which is available to authorized users.

## Background

Acute myocardial ischemia (AMI) is commonly associated with heart disease and frequently manifests in the clinic as pericardial discomfort, or chest pain. Diagnosis of AMI is an important factor in the determination of patient prognosis; however, in cases of AMI without myocardial infarction, echocardiogram examination cannot reveal the abnormal segmental wall activity. Coronary angiography is the most commonly used and accurate technique for atherosclerosis diagnosis, but it is invasive and expensive.

Two-dimensional speckle tracking imaging (2D–STI) is a relatively new method of angle-independent quantification of left ventricular (LV) strain, strain rate and LV twist [1–4]. The technique’s namesake “speckles” are the result of the constructive and destructive interference patterns observed in conventional gray-scale ultrasound images. Tracking the unique speckle pattern from one frame to the next allows the investigator or clinician to track myocardial motion [5].

A normal myocardium consists of endocardium, middle myocardium, and epicardium fibers. Current 2D–STI researches on the myocardial function has primarily focus on the global systolic and/or diastolic function, the torsion and torsion velocity changes between the apex and the base of the heart [6–12]. Few studies have reported the use of 2D–STI to assess the endocardium, middle myocardium, and epicardium of the LV function and longitudinal rotation (LR) of the LV. However, some scholars had noted the different layers of the LV can make its function more clearly [13]. There was clockwise LR during the systolic period in the LV of patients with dilated cardiomyopathy or heart failure [14, 15]. More importantly, most of these reports have described the phenomenon in human patients, and few studies have attempted to investigate LR using animal models.

Therefore, the first aim of our research was the measure LV peak systolic longitudinal strain (LS) of the endocardium, middle myocardium, and epicardium, peak longitudinal strain rate (LSr). Second, we phrased a specific hypothesis which is there have global LR in the LV of rabbits. In this study, we sought to investigate whether LR occurs and evaluated early longitudinal function changes in the LV of rabbits after AMI.

## Methods

This study conformed to the Guide for the Care and Use of Laboratory Animals and was approved by the Ethics Committee of Changzhou No.2 People’s Hospital Institutional Animal Care and Use Committee. The methods carried out in the experiment were in accordance with the approved guidelines. The ARRIVE guidelines (Additional file 1) were followed in this study.

### Animal preparation

Forty-eight New Zealand white rabbits (males: 28 and females: 20), weighing 1.5–2.0 kg, were provided by the Experimental Animal Center of NanJing Medical University, China. All rabbits were lived in an alone cage, that the dimensions were 80 cm for length, 60 cm for width and 60 cm for height. Every day we feed them 3–4 times, and washed the cages 2 times (am: 8:00, and pm: 15:00). All of the rabbits were healthy, and all of them had not examined or treated by veterinarians before or during the research procedures. All rabbits were anesthetized with pentobarbital (1.5%, 2 ml/kg, IP), and ECG leads were connected to each subject. Thoracotomy was performed to expose the heart, and a suture was used to occlude the left anterior descending coronary artery (LAD) for 10 min, following the procedure we just used the Lidocaine to reduce the pain. We used Lidocaine just before thoracotomy and begin to closed thorax. We had taken the subcutaneous injection method. In general, every rabbit used a piece of Lidocaine (5 ml: 0.1 g). Afterwards, the thorax was closed and the rabbits were monitored by ECG to confirm ST segment changes and the establishment of the acute myocardial infarction model.

### Conventional two-dimensional Doppler echocardiography

Conventional echocardiography was performed on forty-eight New Zealand white rabbits using the GE Vivid7 Dimension ultrasound machine. Preoperative and postoperative LV end-systolic volume (LVESV), end-diastolic volume (LVEDV), stroke volume (SV) and LV ejection fraction (LVEF) were recorded in the rabbits, and standard high frame rate apical four, three and two-chamber views of three consecutive cardiac cycles were stored for off-line analysis.

### Data analysis for LR behavior of LV

**Fig. 1:**
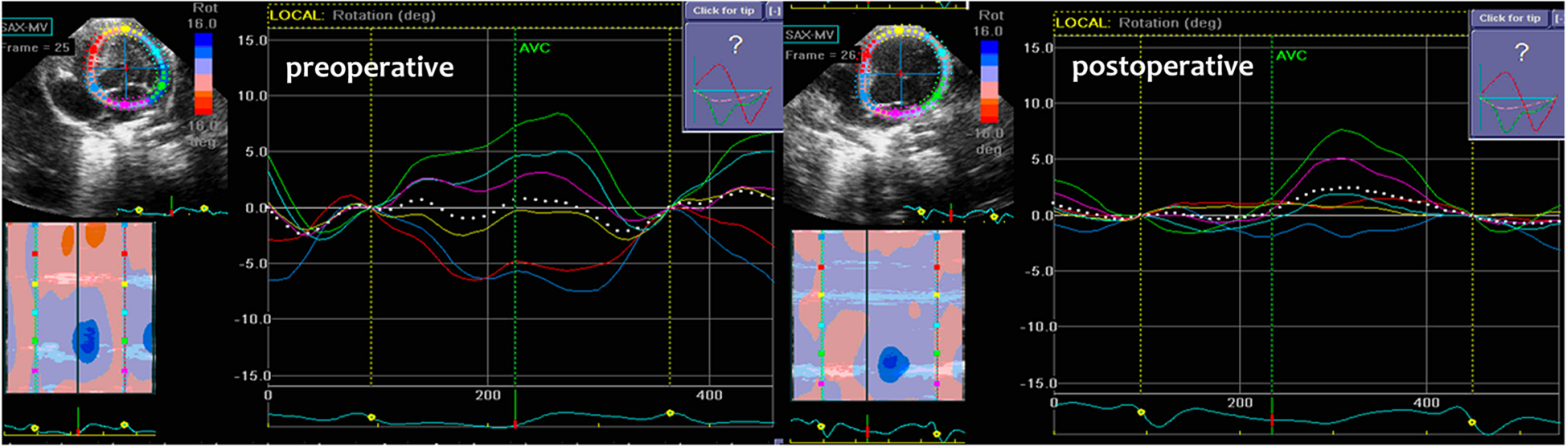
Global LR between preoperative and postoperative, the *left picture* presented the preoperative, and the *right picture* presented the postoperative

We analyzed the apical four, three and two-chamber preoperative and postoperative views using 2D–STI software to obtain the strain, strain rate index and LR (2D–Strain, EchoPAC PC version 113, GE Healthcare, Horten, Norway). LR was defined by the global rotation of the LV cross-section. We sketched the endocardial contours, and the software automatically created regions of interest containing the endocardium, middle myocardium, and epicardium, then adjusted the regions of interest to match the endocardial-myocardial and epicardial-myocardial borders. In the apical four-chamber, three-chamber, two-chamber views, the LV walls were subdivided into six: septum, lateral, anteroseptum, posterior, anterior, inferior walls. Segmental LR and global LR of the LV were assessed from the same echocardiograms using 2D–STI (Fig. 1). All animals were euthanized once data collection was complete, the method of euthanized was injected a certain amount of air (20-50 ml) into the ear vein. All of the rabbits were anesthetized prior to euthanasia via air embolism.

### Statistical analysis

All analysis was performed using SPSS 17.0 (SPSS Inc., Chicago, IL, USA). Normal data distribution was assessed using Kolmogorov-Smirnov’s test. If the data distribution was normal, the data was compared using a paired *t*-test. For variables with non-normal distribution, the nonparametric Mann-Whitney test was used. Data was expressed as the mean ± SD. Differences were considered statistically significant when the *p*-value was less than 0.05.

## Results

### Basic information

The values of postoperative LVEF were significantly lower than the values observed in preoperative rabbits. There were no significant differences between the preoperative and postoperative LVEDV, LVESV and SV (Table 1).

**Table 1:** The basic information of conventional two-dimensional doppler echocardiography between preoperative and postoperative ($\overline{x}\pm \mathrm{s}$)

	HR(bpm)	LVEDV(ml)	LVESV(ml)	SV(ml)	LVEF(%)
Preoperative	276 ± 44	1.61 ± 1.36	0.52 ± 0.19	1.09 ± 1.28	63 ± 9
Postoperative	241 ± 47	1.27 ± 0.43	0.57 ± 0.23	0.70 ± 0.26	55 ± 9
*P* Value	<0.001	0.119	0.248	0.057	<0.001

*HR* heart rate, *LVEDV* left ventricular end-diastolic volume, *LVESV* left ventricular end-systolic volume, *SV* stroke volume, *LVEF* left ventricular ejection fraction

### Compared LS of the endocardial, middle and epicardial myocardial layers between preoperative and postoperative rabbits

**Fig. 2:**
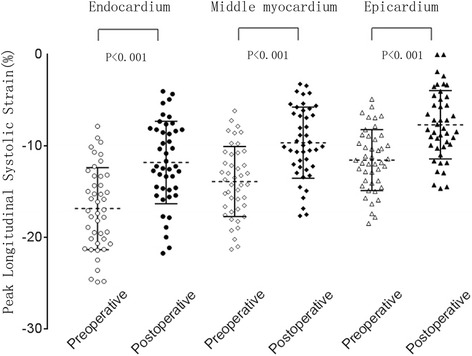
Scatter diagram reveals the peak global systolic longitudinal strain of endocardial, middle and epicardial layers between preoperative and postoperative rabbits

In the preoperative and postoperative rabbits, the pattern of peak systolic LS of the three different myocardial layers was: endocardium > middle myocardium > epicardium. The peak systolic global LS was significantly different between the preoperative and postoperative rabbits. The values of the postoperative were lower than preoperative. The segmental peak systolic LS of the three different myocardial layers had significant difference between the preoperative and postoperative rabbits exclude the epicardial and middle myocardial layers of the septal wall. The values of the postoperative were lower than preoperative. (Tables 2 and 3, Fig. 2)

**Table 2:** The peak global systolic longitudinal strain of endocardial, middle and epicardial layers, peak global longitudinal strain rate between preoperative and postoperative ($\overline{x}\pm \mathrm{s}$)

Varible	Peak global systolic longitudinal strain(%)			Peak global longitudinal strain rate (S^−1^)		
	Endocardial	Middle	Epicardial	Systolic	Early-diastolic	Late-diastolic
Preoperative	−16.84 ± 4.49	−13.88 ± 3.84	−11.53 ± 3.34	−2.59 ± 0.71	2.93 ± 1.03	3.33 ± 0.96
Postoperative	−11.79 ± 4.51	−9.63 ± 3.89	−7.66 ± 3.74	−1.68 ± 0.77	1.65 ± 1.19	2.05 ± 1.03
*P*-value	<0.001	<0.001	<0.001	<0.001	<0.001	<0.001

**Table 3:** The peak segmental systolic longitudinal strain of endocardial, middle and epicardial layers ($\overline{x}\pm \mathrm{s}$)

Segmental LV wall		Endocardial (%)			Middle (%)			Epicardial (%)		
		Preoperative	Postoperative	*P*-value	Preoperative	Postoperative	*P*-value	Preoperative	Postoperative	*P*-value
Apical 4-CH view	Lateral	−15.38 ± 7.90	−8.55 ± 6.58	<0.001	−12.64 ± 6.77	−6.70 ± 5.42	<0.001	−10.61 ± 5.99	−5.32 ± 4.62	<0.001
	Septum	−13.95 ± 7.32	−10.77 ± 7.19	0.026	−11.27 ± 6.15	−9.01 ± 6.08	0.054	−9.25 ± 5.33	−7.71 ± 5.29	0.119
Apical 3-CH view	Anteroseptum	−15.75 ± 7.72	−8.03 ± 7.10	<0.001	−12.28 ± 6.28	−6.35 ± 5.89	<0.001	−9.65 ± 5.26	−5.13 ± 5.00	<0.001
	Posterior	−17.33 ± 6.88	−10.97 ± 8.21	<0.001	−14.01 ± 6.05	−8.84 ± 6.87	<0.001	−11.46 ± 5.44	−7.21 ± 5.88	0.001
Apical 2-CH view	Anterior	−15.49 ± 7.07	−7.51 ± 7.46	<0.001	−12.55 ± 6.42	−5.85 ± 6.16	<0.001	−10.31 ± 5.96	−4.64 ± 5.20	<0.001
	Inferior	−17.34 ± 7.38	−12.90 ± 8.69	0.009	−13.89 ± 6.26	−10.40 ± 7.22	0.013	−11.18 ± 5.44	−8.49 ± 6.15	0.020

### Compared LSr in systolic and diastolic period between preoperative and postoperative rabbits

**Fig. 3:**
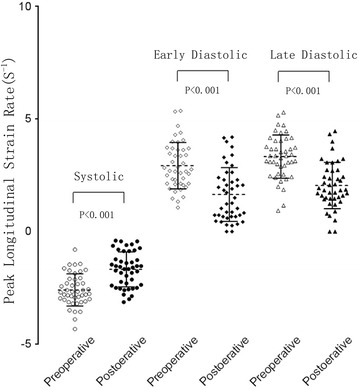
Scatter diagram reveals the peak global longitudinal strain rate between preoperative and postoperative rabbits

The global peak LSr in the systolic, early and late diastolic period had significant difference between the preoperative and postoperative rabbits. The values of the postoperative were lower than preoperative. The segmental peak LSr in the systolic, early and late diastolic period had significant difference between the preoperative and postoperative rabbits exclude the systolic and early diastolic of the septal. The values of the postoperative were lower than preoperative. (Tables 2 and 4, Fig. 3)

**Table 4:** The peak segmental longitudinal strain rate between preoperative and postoperative ($\overline{x}\pm \mathrm{s}$)

Segmental LV wall		Peak longitudinal systolic strain rate (S^−1^)			Peak longitudinal early-diastolic strain rate(S^−1^)			Peak longitudinal late-diastolic strain rate (S^−1^)		
		Preoperative	Postoperative	*P*-value	Preoperative	Postoperative	*P*-value	Preoperative	Postoperative	*P*-value
Apical 4-CH view	Lateral	−3.16 ± 1.19	−2.16 ± 1.27	<0.001	3.51 ± 2.45	1.82 ± 1.77	0.001	4.61 ± 2.58	2.65 ± 2.15	<0.001
	Septum	−2.82 ± 1.27	−2.40 ± 1.25	0.100	2.61 ± 1.59	2.36 ± 1.76	0.417	3.45 ± 1.87	2.67 ± 1.71	0.023
Apical 3-CH view	Anteroseptum	−3.12 ± 1.32	−2.11 ± 1.23	<0.001	2.95 ± 1.72	1.85 ± 1.49	0.004	3.04 ± 1.65	1.93 ± 1.38	0.005
	Posterior	−3.53 ± 1.58	−2.27 ± 1.19	<0.001	3.31 ± 1.94	2.20 ± 1.58	0.006	4.04 ± 1.69	2.58 ± 1.47	<0.001
Apical 2-CH view	Anterior	−2.97 ± 1.27	−2.06 ± 1.25	0.003	3.54 ± 2.15	1.50 ± 2.21	<0.001	4.53 ± 2.39	2.26 ± 1.72	<0.001
	Inferior	−3.39 ± 1.24	−2.58 ± 1.43	0.001	3.35 ± 1.99	2.25 ± 1.89	0.005	4.84 ± 2.46	3.30 ± 2.18	0.001

### Compared segmental LR with global LR in the LV of preoperative and postoperative rabbits

**Fig. 4:**
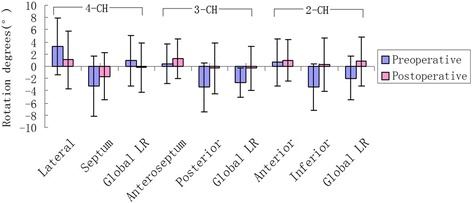
Segmental wall rotation and the global LR between preoperative and postoperative rabbits, *blue* means the preoperative, while the *magenta* means the postoperative

In the apical four-chamber view, there were significant differences in the degrees of rotation of the LV lateral wall in preoperative and postoperative rabbits. In the apical three-chamber view, the rotation degrees of the posterior wall was significantly lower in the postoperative rabbits, and the LR was also significantly lower in the postoperative than in the preoperative rabbits (−2.66° ± 2.34°, −0.33° ± 3.57°, *p* < 0.001). In the apical two-chamber view, the degrees of rotation about the inferior wall was significantly lower in the postoperative than in the preoperative rabbits, and the LR was also significantly lower in the postoperative rabbits (−1.96° ±3.59°, 0.79° ± 3.98°, *p* = 0.004). (Table 5, Fig. 4)

**Table 5:** The segmental and global longitudinal rotational between preoperative and postoperative (°, $\overline{x}\pm \mathrm{s}$)

Segmental LV wall rotational degrees(°)		Preoperative	Postoperative	*P* Value
Apical 4-CH view	Lateral	3.23 ± 4.65	1.07 ± 4.74	0.043
	Septum	−3.27 ± 4.91	−1.64 ± 3.89	0.095
	Global LR	0.94 ± 4.14	−0.21 ± 3.99	0.136
Apical 3-CH view	Anteroseptuml	0.39 ± 3.24	1.28 ± 3.21	0.199
	Posterior	−3.45 ± 4.05	−0.29 ± 4.16	<0.001
	Global LR	−2.66 ± 2.34	−0.33 ± 3.57	<0.001
Apical 2-CH view	Anterior	0.68 ± 3.86	0.93 ± 3.37	0.725
	Inferior	−3.36 ± 3.84	0.29 ± 4.41	<0.001
	Global LR	−1.96 ± 3.59	0.79 ± 3.98	0.004

## Discussion

In the research, we had ligated the left anterior descending coronary artery to make an acute myocardial infarction model. The main findings of the study were: (1) the peak systolic LS of different myocardial layers, peak LSr in the postoperative rabbits were lower than in the preoperative. (2) There was LR in the cardiac of the postoperative rabbits.

AMI is a common heart disease. In recent years, the incidence of AMI and its associated mortality have increased annually. Cell death occurs after approximately 20 min of severe ischemia, and progresses in a wave front from the subendocardium into the subepicardium of the ischemic bed-at-risk. In ischemia, the lack of oxygen is caused by a reduction in coronary flow and towards cell death and necrosis and or infarction [16]. In order to reduce the mortality rate, we performed a 10 min LAD ligation in rabbits. The acute occlusion of the coronary can damage the systolic and diastolic function of the cardiac. From this research, we knew that, the function of the LV damaged after AMI. After knowing this knowledge, fighting for the time to re-open the occlusion artery is becoming more important.

However, early diagnosis of AMI is difficult. While coronary angiography can accurately evaluate the degree of stenosis of coronary artery, the technique is prohibitively invasive and expensive. 2D–STI is a new technique that is useful for the effective diagnosis of myocardial ischemia, myocardial infarction. In acute myocardial infarction, 2D–STI can easily and accurately determine the infarcted area [17–21]. However, few studies have been performed concerning 2D–STI’s ability to diagnose myocardial ischemia in animal models.

Qian FU et al. [22] evaluated changes in the regional LV myocardial function of rats following acute occlusion of the left anterior descending coronary artery using 2D–STI. They concluded that 2D–STI was a non-invasive technique that can be used to assess the response of regional myocardial function to variations in blood supply in rats following acute occlusion of the LAD. They also concluded 2D–STI could be used as a sensitive and reliable means to follow the process of LV remodeling.

An advantage of rabbits as experimental subjects for myocardial ischemia is that rabbits do not require artificial ventilation during the LAD ligation experiments. According to previous studies, rabbits have minimal coronary collateral circulation. Blood flow to the coronary artery of the rabbit is mainly via the left anterior descending coronary artery, LV branch and the right coronary artery. The LAD perfuses the LV and a small portion of right ventricle wall, and the LV branch supplies a wider portion of the LV, but the right coronary artery only perfuses the right ventricular free wall [23]. LAD ligation in the rabbit is, therefore, a good model of AMI.

In our studies, we experimented with several methods of producing an accurate model of AMI in the rabbit. We observed that ligate the root of the LV branch might lead to sudden death. Ligating the distal end of the LV branch did not produce a good model of AMI, and caused papillary muscle and apical myocardial infarction instead. Therefore, we choose to model of AMI by ligating LAD. This study presented an innovative evaluation of the changes in LV function by detecting the three different myocardial layers and LR in the early stages of AMI in rabbits using the 2D–STI technique. We examined LV function and LR in preoperative rabbits as a self-control to minimize the variability between the groups.

The study reported in this article investigated a rabbit model of AMI induced following LAD ligation. We used this model to calculate and compare the LV function. The values of postoperative LVEF were significantly lower than the values observed in preoperative rabbits, and there were no significant differences in the LVEDV, LVESV and SV between the preoperative and postoperative rabbits. These values indicated that the ligation of LAD in rabbits had a significant influence on LV systolic function.

The pattern of peak systolic LS of the three different myocardial layers was: endocardium > middle myocardium > epicardium. At rest, most left ventricular wall thickening occurs as a result of endocardial thickening; the middle myocardium layer contributes only modestly to thickening; and the contribution of the epicardium is negligible [24]. According to the different orientation of myocardial fibres, the wall stress was in a non-uniform distribution, with decreasing values from endo- to epicardium. The subendocardial longitudinal fibres have a smaller curvature as the mid-wall circumferential fibres, also to the subepicardial fibres; thus, the wall stress will be higher on the longitudinal fibres [20]. The Peak systolic LS and LSr in the infarcted, peri-infarcted and remote myocardial regions were significantly decreased in the postoperative. From this results, we concluded that the longitudinal LV function of postoperative rabbits were impaired in the early phase of AMI.

This study used the LR of the LV to reflect changes in systolic function following AMI in rabbits. Previous studies have reported LR in the LV of dilated cardiomyopathy patients and other heart failure patients; however, before our study it was unknown whether LR was associated with AMI in rabbits. We observed that the degrees of rotation of the LV lateral, posterior and inferior walls were significantly lower in the postoperative rabbits (*p* < 0.05), and the LR in the apical three and two-champer views were also significantly lower (−2.66° ± 2.34°, −0.33° ± 3.57°, *p* < 0.001, −1.96° ±3.59°, 0.79° ± 3.98°, *p* = 0.004). We concluded that the LR changes between the preoperative and postoperative rabbits were related to the anatomy of rabbit coronary.

The LAD of the left coronary artery perfuses the interventricular septum, and when it is occluded, the LR of interventricular septum had no significant differernce between preoperative and postoperative rabbits, but the value is decreased. For the reason, we considered it was related to the collateral circulation, when the LAD was occluded, the collateral circulation maybe rebuilded. Why, then, were the LR of the inferior and posterior walls reduced? Due to the “muscle band”, the inferior and posterior walls are downstream of the interventricular septum, and, therefore, they may be influenced by the upstream muscle. Our results demonstrated that LR may be related to the condition of the surrounding muscle. The origin of the LR is unclear. Gallagher KP et al. [25] performed a research in conscious dogs, and the found that inner and outer wall thickening during systole is not uniform and the nonuniformity is sustained during the greatly altered hemodynamic conditions associated with exercise. After the acute occlusion of the coronary of LAD, according to the distribution of the myocardial fibre, the subendocardium was affected the most. When the cardiac contracted, due to AMI, the original balance of the different myocardium layers was disappeared or changed, may resulting in the changes of LR motion. Also the global LR reversed in the apical 4-ch and 2-ch views maybe explained by the above reason. By examining the LR, it can be determined that early AMI in the LV influenced the function of the heart.

## Conclusions

In this study, LV function was impaired in the early phase after acute occlusion of LAD coronary artery. The LS of different myocardial layers could reflect the LV function very conveniently. The LR of LV could be an important indicator of cardiac function in the early phases of AMI. In the clinical practice, the early detection of the cardiac dysfunction after AMI can make these patients have early treatment, and can also evaluate the efficacy of different treatment.

## Additional file

Additional file 1: The ARRIVE Guidelines. (PDF 258 kb)

## Abbreviations

2D–STI: 2-dimensional speckle tracking imaging
AMI: Acute myocardial ischemia
LAD: Left anterior descending artery
LAD: Left anterior descending coronary artery
LR: Longitudinal rotation
LS: Longitudinal strain
LSr: Longitudinal strain rate
LV: Left ventricular or left ventricle
